# What's Hot and What's New in Xenotransplantation From the Young Investigator Committee of the IXA–Basic and Translational Science

**DOI:** 10.1111/xen.70150

**Published:** 2026-06-14

**Authors:** Corbin E. Goerlich, Evelyn J. Salvaris, Konrad Fischer, Antonio Citro, Alessia Giarraputo, Joseph Ladowski, Emilien Seizilles de Mazancourt, Liaoran Wang, Daniel Eisenson, Margaret R. Connolly, Jeffrey Stern, Alban Longchamp, Raphael P. H. Meier

**Affiliations:** ^1^ Stanford Hospital Palo Alto California USA; ^2^ Immunology Research Centre St Vincent's Hospital Melbourne Victoria Australia; ^3^ Institute of Surgical Research, Walter Brendel Center of Experimental Medicine, University Hospital LMU Munich Munich Germany; ^4^ San Raffaele Diabetes Research Institute, IRCCS San Raffaele Scientific Institute Milan Italy; ^5^ Department of Pathology Massachusetts General Hospital and Harvard Medical School Boston Massachusetts USA; ^6^ Department of Surgery Duke University School of Medicine Durham North Carolina USA; ^7^ Paris Institute of Transplantation and Organ Regeneration Paris France; ^8^ Second Affiliated Hospital of Hainan Medical University Haikou China; ^9^ Johns Hopkins Hospital Baltimore Maryland USA; ^10^ Heart and Vascular Center, Brigham and Women's Hospital Boston Massachusetts USA; ^11^ Department of Surgery NYU Langone New York New York USA; ^12^ Department of Surgery, Division of Transplant Surgery Massachusetts General Hospital, Harvard Medical School Boston Massachusetts USA; ^13^ Department of Surgery, Division of Transplant Surgery University of Maryland School of Medicine Baltimore Maryland USA

## Abstract

Xenotransplantation has been iteratively improved over the last decade in pre‐clinical models, with first‐in‐human clinical trials underway. The 2025 IXA Congress in Geneva was held in parallel with meetings involving World Health Organization leaders to support the development of new guidance on xenotransplantation in this rapidly evolving field. Key scientific themes of the meeting included the introduction of multiple‐gene edited pigs for xenotransplantation research and clinical trials, better characterization of innate and adaptive immune responses and xenograft preservation that optimizes ramifications of ischemia‐reperfusion injury during implantation. This comes at a time when gene‐edited pig organs are being used for human xenotransplantation, a development that demands safety, reproducibility, durability, and a clear mechanistic understanding of rejection and tolerance.

## 1 Introduction

Recent years have seen a convergence of genetic engineering, immunology, and transplantation biology that is transforming our understanding of xenotransplantation. Since the 2023 International Xenotransplantation Association (IXA) Congress, these advances have contributed to progressively longer graft survival. Across multiple organs in preclinical research, investigators highlighted how iterative refinement of donor genetic modifications, immune modulation and tolerance strategies, as well as organ preservation continue to reveal the fundamental of xenograft compatibility and long‐term function. The 2025 IXA Congress in Geneva was held in parallel with meetings involving World Health Organization leaders to support the development of new guidance on xenotransplantation.

**FIGURE 1:**
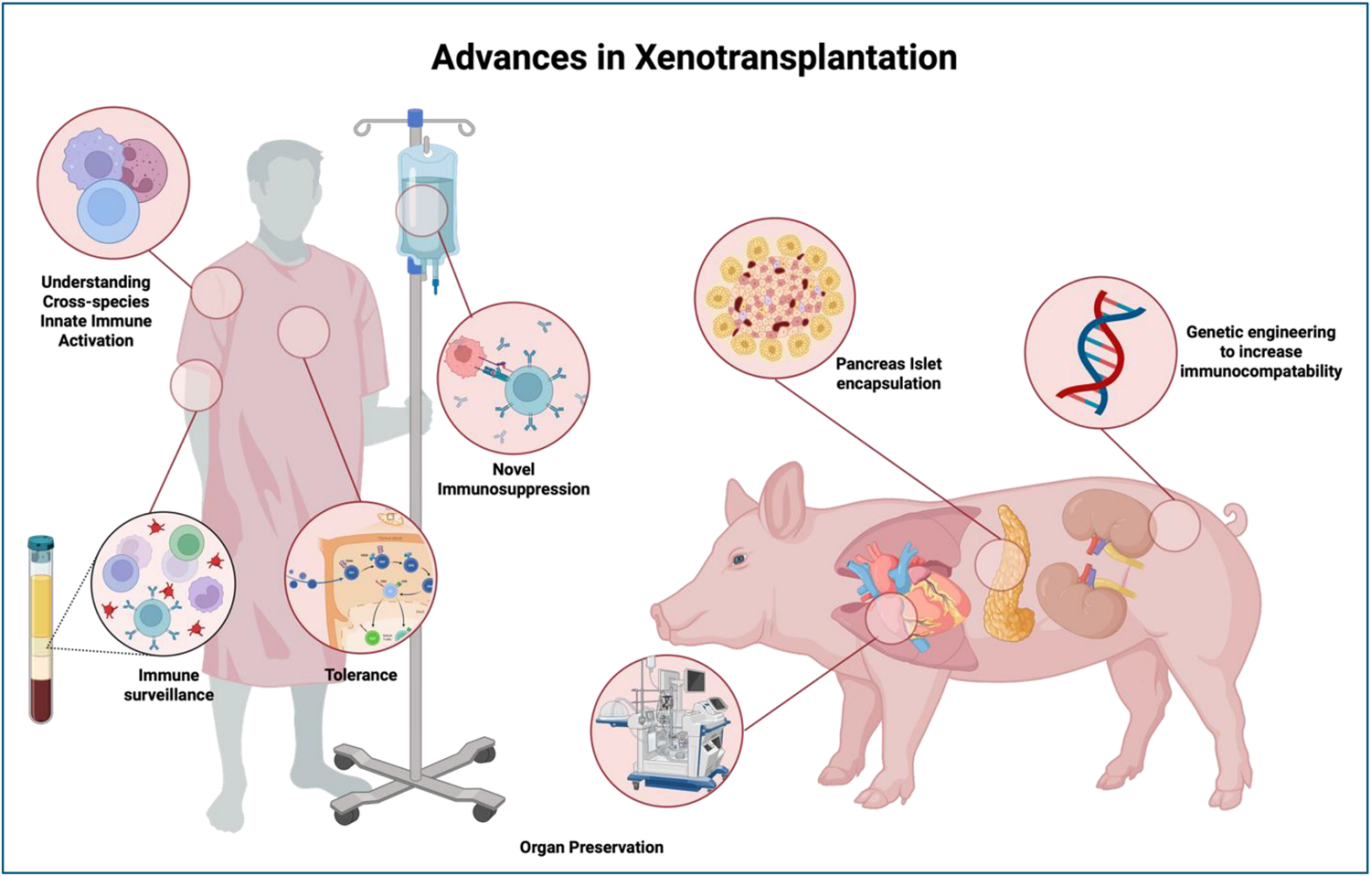
Advances in Xenotransplantation. Xenotransplantation has seen significant, multi‐faceted progress, as reflected in work presented at the International Xenotransplantation Association (IXA) 2025 Congress in Geneva.

The following synthesis summarizes six major scientific themes that emerged from the meeting (Figure 1): (1) next‐generation humanized, clinically ready gene‐edited donor pigs; (2) experimental tolerance mechanisms achieved through thymokidney composite grafts; (3) high‐resolution immunologic profiling of (pre) rejection; (4) the innate immune interface (particularly NK‐ and myeloid‐cell activation pathways) leading to early xenograft failure; (5) strategies and mechanism of storage induced xenograft injury; and (6) recent progress in islet xenotransplantation and bioengineering. Collectively, these advances illustrate how basic and translational research continues to provide the framework and necessary evidence for clinical trials. Congress abstracts are compiled in the published IXA 2025 abstract supplement and are referenced below by abstract number [1].

## 2 Genetic Engineering and Donor Design

Recent progress in xenotransplantation is driven by increasingly sophisticated genetic engineering of donor pigs, aimed not only at overcoming innate immunologic barriers, but also at addressing hematologic complications and ischemia–reperfusion injury. Across several studies, a coherent picture is emerging in which antigen deletion, insertion of human immunomodulatory genes, and improved donor platforms are converging to make pig‐to‐primate (and ultimately pig‐to‐human) transplantation increasingly feasible [2]. Genetic engineering is also expanding to novel targets (e.g., hematopoiesis), while streamlining workflows for high‐throughput production of genetically modified pigs.

Expression of human erythropoietin demonstrates that donor organs can be engineered to mitigate posttransplant anemia, a common complication after kidney transplantation. Kidneys from EPO‐transgenic pigs maintained stable hemoglobin levels in rhesus macaques without requiring recombinant EPO supplementation (106.1). While the clinical relevance of this approach remains to be defined, particularly given that erythropoietin can be readily supplemented and is not universally required in all patients, these findings illustrate the potential of genetic engineering to modulate systemic physiological functions. Of note, in one of the clinical cases, erythropoietin was initiated on day 15 after transplantation [3]. Additionally, triple‐knockout red blood cells engineered to overexpress human CD55 exhibited reduced human IgM/IgG binding and complement‐mediated hemolysis, approaching compatibility levels similar to human RBCs in vitro. Together, these strategies highlight how genetic modifications can render porcine‐derived organ and cellular blood‐support systems compatible in humans.

A second thematic cluster involves building improved donor pigs and gene‐editing pipelines. Auckland Island pigs have emerged as a promising donor breed owing to their smaller size, normal cardiac anatomy, and PERV‐C–free status (106.2). These animals also display complete homozygosity at the SLA locus, simplifying immunologic matching. On this foundation, a minimal genetic modification set (*GGTA1* knockout with human CD46 and thrombomodulin) was introduced and demonstrated ubiquitous transgene expression. Complementary advances in gene editing include a modular CRISPR/Cas9–Cre/lox recombinase–mediated cassette exchange platform enabling targeted insertion of six human transgenes into the *GGTA1* locus in a single step. Comprehensive screening across preclinical and clinical settings continues to show no evidence of PERV transmission, supporting the virological safety of current xenotransplantation approaches. These findings reinforce the regulatory feasibility of clinical translation, although continued surveillance remains essential (212.3). Porcine expanded potential stem cells (EPSCs) extend this further by providing a highly editable, stable platform for complex modifications (111.3). Quadruple‐knockout EPSCs combined with ROSA26‐targeted human CD47 produce endothelial cells with reduced antibody binding, complement injury, and phagocytosis, illustrating the potential for rapid generation and testing of candidate donor genotypes.

A novel target for modulating immunocompatibility is the manipulation of swine leukocyte antigen and stress‐response pathways. SLA‐deficient pigs lacking class I and II molecules and expressing multiple human complement‐regulatory genes show reduced human T‐cell activation in humanized mice and extended survival in nonhuman primate kidney transplantation, especially with anti‐CD154 therapy (106.3). Supporting this, SLA‐deficient pigs exhibit significantly decreased human and primate CD8^+^ T‐cell proliferation, reinforcing SLA's contribution to delayed xenograft rejection (111.4). At the innate interface, human and porcine HO‐1 transgenes similarly protect endothelial cells from monocyte‐, neutrophil‐, and NK‐cell–mediated cytotoxicity. This is particularly relevant in light of emerging evidence highlighting a central role for NK cells in xenograft rejection in humans [4, 5].

Finally, the AKIP1 transgenic pig model introduces a mechanistically targeted approach to attenuating oxidative stress and mitochondrial injury, central drivers of ischemia–reperfusion damage. These pigs show reduced apoptosis, necrosis, and ferroptosis, alongside preserved mitochondrial function after oxidative stress. This represents a unique advance, directly addressing organ injury pathways that limit graft longevity [6].

## 3 Evidence of Tolerance From Thymokidneys and Immunologic Assessment of Immune Tolerance

The clinical translation of composite thymokidney xenografts represents an approach to achieving donor‐specific immune tolerance in xenotransplantation. Dr. Sykes et al. reported the first human evidence of functional thymopoiesis within a porcine thymus graft, in which a 54‐year‐old woman at NYU received a *GGTA1*‐knockout thymokidney. The graft demonstrated immunologic reconstitution: T‐cell counts recovered by postoperative day 28, with 64% of CD4 cells exhibiting the CD45RA^+^CCR7^+^CD31^+^ phenotype characteristic of recent thymic emigrants, an increase from <10% pretransplant. Histologic examination of the explanted graft revealed human CD45^+^ leukocytes comprising 0.85% of thymic cells, including an immature thymocyte population. Functional immunologic assays demonstrated progressive donor‐specific hyporesponsiveness in mixed lymphocyte reactions by days 47 and 86, with preserved reactivity to third‐party antigens, providing proof‐of‐concept for tolerance induction through xenogeneic thymopoiesis in a human recipient (205.1).

However, there may be limits to thymus‐dependent tolerance in xenotransplantation, as demonstrated in systematic immunosuppression‐withdrawal studies in nonhuman primate (NHP) models. Gunes and colleagues documented that baboons receiving GalT‐KO, SLA^hh^ thymokidneys developed robust recent thymic emigrant populations and sustained donor‐specific hyporesponsiveness. Gradual drug tapering after 6–13 months of stable function enabled maintenance on belatacept monotherapy for more than two months in both subjects. Analysis via CD25‐depleted mixed lymphocyte reaction confirmed Treg involvement in one animal's hyporesponsiveness. Nonetheless, both eventually experienced acute rejection, indicating that current thymic grafts enable substantial but incomplete immunosuppression reduction. These results highlight the need for refined tolerance protocols to modulate allo‐ and xeno‐immune responses and improve long‐term graft outcomes (205.4).

Complementing these clinical and experimental findings, mechanistic studies have elucidated optimal strategies for tolerance induction. Mcheik and colleagues demonstrated that transplantation of dual pig and human thymus, combined with mixed‐chimerism induction, overcomes critical limitations inherent to single‐thymus approaches. T cells educated solely within pig thymus lack recognition of human tissue‐restricted antigens and exhibit suboptimal peripheral homeostasis. The dual‐thymus strategy enables complementary selection: human thymic tissue eliminates autoreactive clones and generates tissue‐specific regulatory populations, while porcine thymus produces SLA‐restricted cells and pig‐specific Tregs. Crucially, depletion experiments revealed predominantly deletional rather than regulatory mechanisms underlying tolerance, suggesting durability independent of ongoing Treg suppression. This advancement supported the achievement of tolerance without thymectomy, substantially enhancing clinical feasibility and application (205.2).

In this framework, complementary approaches targeting local microenvironments may augment systemic tolerance mechanisms. One strategy is to engineer mesenchymal stem cells to overexpress CCL22 to reshape immune responses at transplant sites. Through CCR4‐mediated recruitment, these cells enriched regulatory T‐cell populations while promoting IL‐10 and TGF‐β secretion. Interestingly, subcutaneous implantation drove macrophage polarization from pro‐inflammatory (M1) to immunoregulatory (M2) phenotypes. A similar strategy has been explored using engineered MSCs to enhance graft revascularization and function, for example through MMP‐9 overexpression, which improves local microenvironment remodeling, promotes vascularization, and supports xenograft survival in islet transplantation models (211.6). Although validated in allogeneic rather than xenogeneic contexts, localized chemokine‐directed immunomodulation could synergize with thymus‐induced systemic tolerance (205.5).

Finally, the translational potential and readiness of thymokidney xenotransplantation, even with a single‐knockout design, has been further validated by the first Good Laboratory Practice (GLP) preclinical trial of pig‐to‐baboon thymokidney transplantation. The Columbia group achieved consistent one‐year xenograft survival in baboons receiving single GalT‐KO thymokidneys from miniature swine donors with standardized immunosuppression. This rigorous GLP study meets the International Xenotransplantation Association's recommendation for preclinical studies demonstrating consistent survival exceeding six months. Recent thymic emigrants emerged within two months across all recipients, and four animals developed measurable donor‐specific hyporesponsiveness. Renal function remained stable throughout the study period, with histology demonstrating minimal transplant glomerulopathy and no evidence of cellular or humoral rejection. These findings support a favorable and independent immunomodulatory role of the thymic component within thymokidney composite grafts (310.3).

Recent evidence shared during the IXA Congress supported that composite thymokidney grafts can support functional human thymopoiesis, induce measurable donor‐specific hyporesponsiveness, and allow marked immunosuppression reduction. Several questions remain for further study, including the optimal degree of genetic modification required when thymic tissue is encapsulated and whether immunosuppressive drug reduction is achievable with current constructs. Standardized monitoring frameworks, including recent thymic emigrant assessment, donor‐specific MLR, Treg functionality assays, and longitudinal antibody surveillance, now provide validated tools for rigorously evaluating tolerance in clinical xenotransplantation trials.

## 4 New Insights in the Innate Immune System and Xenotransplantation

As the number of clinical xenotransplantation studies increases, we are gaining insight into the mechanisms underlying xenograft failure. The IXA 2025 conference provided focus and clarity on the role of the innate immune system in xenograft rejection, specifically NK cells, neutrophils, and macrophages. While NK cell–mediated cytotoxicity and neutrophil/macrophage activation and phagocytosis of pig cells are beyond the scope of this review, we aim to succinctly capture key discussions from the field's premier xenotransplantation conference. Three themes merit particular emphasis: (1) foundational research implicating innate cells in xenotransplantation, (2) contemporary methods confirming their presence in clinical xenograft recipients, and (3) emerging approaches to prevent their activation. Recent studies identify Plexin‐B2 expression on porcine endothelial cells as a mechanism to suppress neutrophil‐mediated cytotoxicity, underscoring the role of neutrophils as an underrecognized barrier to xenograft survival. Targeting these pathways may offer additional avenues to improve early graft outcomes (307.4).

In a session dedicated to cellular rejection via innate/adaptive immune responses, Seebach, et al. (200.1) reviewed his group's (and others’) prior in vitro and ex vivo perfusion studies supporting a role for NK cells in xenograft rejection. He highlighted two dominant mechanisms by which NK cells may elicit injury: direct cellular cytotoxicity via class I MHC mismatch (or absence, in the case of the pig) and antibody‐dependent cellular cytotoxicity (ADCC). He concluded by summarizing perfusion models designed to replicate physiologic vascular flow conditions, which have enabled mechanistic studies of innate‐cell rolling, adhesion, and binding strength. Dr. Miyagawa (200.2) summarized macrophage and neutrophil immunology relevant to xenotransplantation, including activation and differentiation pathways discussed elsewhere in this manuscript.

Prior to the first clinical xenograft studies, the contribution of innate immunity to xenograft rejection was largely unproven. However, we now know that innate immune cells play a significant role in the acute post‐xenograft phase. International speakers reported results from bulk, single‐cell, and nuclei RNA sequencing of biopsy samples obtained from living and decedent cardiac and kidney xenograft recipients (Shetty, et al. 105.3, Loupy, et al. 101.3, Schmauch, et al. 200.4). Similar patterns have been reported in liver and lung NHP models (Zhang, et al. 312.1; Dufault, et al. P2.10; Ramm, et al. P2.18; Takemoto, et al. P305.5). Collectively, these data depict an impressive array of assays and multiplex approaches showing substantial infiltration of innate cells and upregulation of innate‐associated markers (e.g., Fc receptors) within 1–3 days post‐xenotransplantation. These cells appear to drive early microvascular inflammation (Loupy, et al. 101.3) as well as late fibrotic remodeling (Goutaudier, et al. 307.2; Shetty, et al. 105.3; Jung, et al. P2.32), which are hallmarks of eventual xenograft failure. Spatial transcriptomic analyses of pig‐to‐human xenografts identified human macrophages as the predominant infiltrating immune population, with evidence of xenoantibody‐mediated rejection driven largely by innate immune pathways. These findings provide high‐resolution insight into the cellular and molecular architecture of rejection and support the central role of multimodal diagnostics in defining immune–graft interactions (312.2).

Recent preclinical data demonstrate that complement activation remains a dominant driver of xenograft injury despite the presence of human complement regulatory transgenes, with loss of hCD55 expression observed in failing grafts. Notably, standard complement inhibitors were ineffective across species, whereas targeted C5 inhibition enabled sustained graft function, underscoring the need for species‐compatible complement blockade strategies (310.4).

Approaches using glycan modification to recruit endogenous complement regulators, such as factor H, demonstrate enhanced resistance of porcine endothelial cells to complement‐mediated injury. This strategy represents a potential adjunct or alternative to transgene‐based protection, expanding the toolkit for controlling complement activation in xenotransplantation (307.2).

Emerging presented data indicate that porcine immune mediators can induce unexpected pro‐inflammatory responses in human immune cells, exemplified by porcine SP‐D promoting macrophage activation rather than immunoregulation. Such species‐specific discrepancies highlight persistent innate immune barriers that extend beyond current genetic engineering strategies (P2.19).

Given the clear role of innate cells in xenograft rejection, a central question is how their activation and downstream injury can be prevented. This is an area of active investigation, with in vitro and in vivo studies supporting roles for HLA‐E, HLA‐G, CD47 (including mutant CD47), and heme oxygenase (HO) expression. Transgenic expression of HLA (e.g., HLA‐E or HLA‐G) provides inhibitory signals that can attenuate NK‐cell activation, and further evidence was presented at IXA by multiple groups (Schulz, et al. 311.1; Dufault, et al. 311.1; Takemoto, et al. 305.5; Seebach, et al. 200.1). Similarly, HO transgene expression has been considered for many years in pig xenograft donors; Yanagino et al. presented data supporting its inclusion (200.2). They compared pig versus human HO‐1, HO‐2, and HO‐3 expression and concluded that integration and expression of human HO genes effectively suppress innate‐cell activation. Additional strategies include expression of plexin‐B2 (Matsumura, et al. 307.4), expression of CD47 (Tran 311.3), and blockade of sialoadhesion and GPIb glycoproteins (Takemoto, et al. 311.2). While several of these transgenes and strategies have already been incorporated into clinical xenograft studies, these data provide further support for their continued use.

In terms of adaptive immunity, a large body of work has been done and immunosuppression protocols tailored toward antibody‐mediated rejection have been performed, setting the basis for immunosuppression protocols today. A large retrospective analysis of 50 pig‐to‐non‐human primate heart transplants highlights the evolving interplay between genetic modifications and immunosuppressive strategies in determining graft survival. These data emphasize that the relative contribution of individual edits versus immunomodulation remains incompletely defined and requires continued systematic evaluation (310.5).

## 5 Cold Static Storage versus Machine Perfusion in Heart/Kidney Transplants and Prospects for Lung/Liver Xenotransplantation

As genetically engineered porcine organs progress toward clinical xenotransplantation, questions surrounding preservation, transport logistics, and susceptibility to ischemia–reperfusion injury (IRI) have gained renewed interest. Because donor animals will be raised in specialized facilities and grafts transported over long distances, understanding the mechanistic consequences of cold/warm ischemia, and the potential benefits of machine perfusion, is essential. Emerging data suggest that porcine organs, particularly from genetically modified donors, may exhibit heightened vulnerability to IRI, with implications for graft viability and early function.

Early mechanistic work explored the effects of prolonged static cold storage (SCS) in kidneys from *GGTA1*‐knockout pigs. Using a normothermic reperfusion model to exclude immune confounders, two days of SCS did not result in overt macroscopic or histologic injury, and urine production resumed upon reperfusion. However, *GGTA1*‐KO kidneys exhibited significantly higher arterial vascular resistance compared with wild‐type controls, suggesting subclinical endothelial or microvascular susceptibility despite preserved structural appearance [7]. These findings indicate that SCS may mask early functional impairment that only becomes apparent under physiologic flow and pressure.

More recently, a comparative study highlighted the profound impact of preservation modality on xenograft survival following transplantation into NHPs [8] Porcine kidneys from GalTKO or GalTKO‐hCD55 donors preserved by SCS for five hours underwent hyperacute rejection within 90 min of reperfusion, even in recipients with low preformed anti‐pig antibodies. Despite appearing grossly intact immediately after revascularization, SCS kidneys rapidly developed mottling, diffuse discoloration, and anuria. In contrast, kidneys preserved by hypothermic machine perfusion (HMP) reperfused without signs of acute injury and maintained function for more than 14 days. Mechanistically, these observations suggest that SCS exacerbates endothelial injury and complement activation in xenografts. These processes are mitigated, though not eliminated, by continuous perfusion.

To date, oxygenated hypothermic perfusion, widely adopted in clinical allotransplantation, has not been systematically evaluated for porcine‐to‐primate kidney xenotransplantation. Given the sensitivity of porcine vascular endothelium to IRI and complement‐mediated damage, oxygenated approaches may offer additional benefit and warrant focused investigation.

In cardiac xenotransplantation, work by Langin et al. [9] provided some of the most compelling mechanistic contrasts between ischemic and perfused preservation strategies. Hearts preserved using static ischemic cardioplegia showed profoundly depressed systolic function after transplantation, accompanied by increased lactate production, impaired oxygen extraction, elevated troponin and transaminases, and, in several cases, rapid development of perioperative cardiac xenograft dysfunction. Conversely, hearts maintained by cold, oxygenated, non‐ischemic continuous perfusion exhibited preserved systolic function, stable metabolic parameters, and absence of early graft failure. These data strongly support continuous perfusion as a means of maintaining myocardial energetics and preventing early IRI‐driven graft loss. Single‐nucleus RNA sequencing analysis by this group has further characterized these findings during the conference (presented, but not published).

For lungs, hypothermic perfusion has increasingly been adopted as a strategy to improve graft viability in transgenic porcine donors, as highlighted at the IXA meeting in Geneva (P2.17). Similarly, extracorporeal liver cross‐circulation models using human decedents demonstrated preserved hepatic architecture after 72 h of normothermic support, despite early transaminase elevation and progressive IgM deposition, providing mechanistic insight into endothelial activation and platelet consumption during xenogeneic interaction (305.1). In this context, genetically engineered porcine livers incorporating triple glycan knockout, multiple human transgenes, and PERV inactivation have been shown to sustain core metabolic functions following xenoperfusion, including bile production and regulation of lactate, ammonia, and coagulation parameters, even in the setting of recipient hepatectomy. These findings support the concept that targeted genetic modifications can partially overcome cross‐species metabolic incompatibilities and enable extracorporeal xenogeneic liver support under limited immunosuppression [10].

Extending these observations to a clinical setting, the first reported case of auxiliary porcine liver xenotransplantation in a living human demonstrated that a 10‐gene‐edited graft can achieve transient metabolic integration, as evidenced by bile production and partial correction of coagulation parameters in the absence of hyperacute rejection. However, graft explantation on postoperative day 38 due to thrombotic microangiopathy underscores the persistence of species‐specific dysregulation of coagulation and endothelial compatibility. Despite this limitation, recipient survival to 171 days provides proof‐of‐concept that xenogeneic liver grafts can deliver clinically meaningful, time‐limited support, while highlighting coagulation imbalance as a central barrier to durable function [11].

Notably, recent clinical application of ex vivo perfusion using a genetically modified porcine liver has provided preliminary evidence supporting the feasibility of Xeno‐liver support as a bridging therapy. In this case, a patient with acute‐on‐chronic liver failure underwent extracorporeal circulation through a gene‐edited pig liver for 66 h, during which stable perfusion, bile production, and improvement in liver function parameters were observed. The patient was subsequently bridged to successful liver allotransplantation [12]. While still at an early stage, this approach highlights the potential of combining machine perfusion technologies with genetically engineered organs to expand therapeutic options for end‐stage liver disease.

Collectively, these studies highlight a consistent theme: porcine xenografts exhibit pronounced sensitivity to ischemia and reperfusion, and machine perfusion (whether hypothermic or normothermic) appears to mitigate early injury more effectively than conventional SCS. As clinical translation accelerates, optimizing organ preservation will be essential to ensure graft viability during transport and to limit early IRI‐driven graft failure.

## 6 Islet‐Cell Progress and Bioengineering Hurdles in Islet Xenotransplantation, and Advantages Over iPSCs

A major step forward in β‐cell replacement therapy has emerged from advances in alternative β‐cell sources, including human iPSC‐derived cells and porcine islets. A recent breakthrough was reported in a first‐in‐human study in which stem‐cell‐derived islets were transplanted into the portal vein of a subject with type 1 diabetes (T1D) under an immunosuppressive regimen, demonstrating graft survival and sustained C‐peptide production over one year [13]. In parallel, progress in genome‐edited β cells has reached clinical testing in primary human islet transplantation: human hypoimmune β cells (HLA class I and II knockout with hCD47 overexpression) were transplanted into T1D recipients, with C‐peptide detected three months after implantation [14]. These findings support the potential of human iPSC‐derived cells and genome‐edited human islets as donor β‐cell replacement sources. However, Ludwig, et al. (303.2) emphasized ongoing challenges associated with stem‐cell‐derived therapies and underscored the advantages of xenotransplantation.

In this context, both adult and neonatal xeno‐islets represent promising alternatives due to scalable production and the amenability of pigs to strategic genome editing. Additional advantages include (i) enhanced safety through encapsulation technologies, gene editing, and designated pathogen‐free pig housing to minimize xeno‐viral risks, and (ii) an unlimited, on‐demand islet supply. Currently, porcine islets offer greater flexibility across β‐cell replacement platforms.

At IXA 2025 (Geneva), multiple groups presented encouraging results using encapsulated wild‐type (WT) pig islets to mitigate immunologic responses. Park, et al. (303.4) demonstrated that WT pig islets infused directly into the livers of baboons receiving systemic immunosuppression and Treg therapy achieved long‐term survival exceeding 800 days. The key determinant of long‐term WT islet graft function was the immunosuppressive regimen with preclinical studies examining clinically applicable regimens—in progress.

Conversely, several groups focused on non‐encapsulated genetically modified pig islets (Ludwig, et al. 303.2). Hawthorne and colleagues (211.5) infused streptozotocin‐induced diabetic baboons with genetically engineered pig islets incorporating hCD55, hCD59, and GalTKO. Their findings highlighted the need for vigilant immune monitoring to enable rapid adjustment of immunosuppression and prevent rejection. Follow‐up work incorporating diliximab, an anti‐CD2 monoclonal antibody transgene, on the same pig background showed normal islet function when transplanted into immunosuppressed baboons and demonstrated local diliximab expression on xenograft biopsies (206.3). However, whether the level of local transgene expression is sufficient to protect the xenograft from T‐cell–mediated rejection is under investigation.

Bottino and colleagues (303.3) emphasized that patients with diabetic nephropathy rarely receive human islet transplants due to the absence of a dedicated waiting list. Bottino supported composite islet–kidney transplantation to simultaneously treat diabetes and end‐stage kidney disease, with the added benefit of prevascularizing islets beneath the donor pig kidney capsule. In a collaborative pilot study (Revivicor–United Therapeutics–MGH–Harvard), neonatal pig islets containing 10 gene edits were transplanted under the kidney capsule of immunosuppressed baboons. Early findings confirmed engraftment and insulin secretion two months posttransplantation. Iwase et al. (206.2) demonstrated six months of normoglycemia in a life‐supporting pig‐to‐baboon model using GalTKO.hCD55 pig islets infused into the liver following transplantation of a donor kidney and a vascularized thymic lobe of matching genotype.

In contrast, encapsulated collagen–alginate devices containing InsuGLP‐1M3R transgenic neonatal pig islets demonstrated improved insulin secretion compared with human islets. When transplanted into immunocompromised streptozotocin‐induced diabetic rats without immunosuppression, normoglycemia was restored and maintained for six months, with minimal IgM/IgG responses at 30 days posttransplant. However, glucagon (but not insulin) was detectable by immunohistochemistry (Mourad et al., 311.6).

Additional studies centered on therapeutics aimed at reducing hypoxia‐induced graft loss (e.g., DA‐1 from Wu, 206.5) and attenuating inflammatory, fibrotic, and immune responses (e.g., IL‐13 from Brayman). Ma, et al. (206.1) described a humanized mouse model developed to evaluate therapeutic candidates and characterize rejection phenotypes and hypoimmune cells. Brayman, et al. (211.6) presented work using microporous annealed particles that showed enhanced engraftment, protection from inflammatory responses, and improved graft survival. In combination with pig islets (WT or genetically modified) or stem‐cell‐derived sources, these adjunct therapies may further improve graft engraftment, survival, and function.

## 7 Discussion

While donor genetic modifications are at the forefront of xenotransplantation, there are some limitations to what genetic modifications can be employed without risking donor pig viability. For example, current approaches to generating SLA I/SLA II knockout pigs may result in markedly immunocompromised animals that cannot be bred under normal agricultural housing conditions and therefore will not be suitable for meeting the high (and affordable) demand for donor organs. Moreover, there are ongoing obstacles to generating animals from EPSCs (chromosomal stability, rearrangements, and practical scalability). However, stable clone donors are currently being bred together to enable stable and self‐sustaining colonies.

Additionally, the optimal immunologic therapy and tolerance strategies to avoid antibody‐ and cell‐mediated rejection in xenotransplantation have yet to be resolved. CD‐40/CD‐40L mAb therapy has shown preclinical efficacy for long‐term kidney, heart, and islet cell xenotransplants. However, recent cases of xenotransplantation in humans have used traditional clinical immunosuppression with limited success, reopening the discussion in light of prior non‐human primate data suggesting otherwise. Tolerance studies have also demonstrated a possible clinically viable approach to xenotransplantation with thymokidney and thymoheart transplants, whereas prior non‐human studies have shown some success with less‐clinically applicable regimens (such as whole‐body radiation). As a case in point, recent success in pediatric clinical allotransplantation has demonstrated real‐world long‐term rejection‐free survival using this strategy in heart transplantation [15].

While advances in iPSC‐derived human beta cells continue to develop, pre‐clinical islet cell xenotransplantation using non‐human primate models demonstrate the potential of porcine islets to restore durable glycemic control. As of now a major clinical limitation still remain the overall number of xeno‐islet required to fully restore normoglycemia, however, advances in genome‐editing strategies continue to enhance immunocompatability and refine islet mass equivalent required to clinical levels, strengthening and advancing the translational pathway of xenogenic islet transplantation towards future clinical evaluation.

## 8 Conclusion

Overall, the major themes from IXA 2025 reinforce that clinical xenotransplantation will be enabled not by any single breakthrough, but by integrated solutions that align donor breeding, genetic engineering, immune control, and organ preservation technologies along with standardized immunologic monitoring. The field is increasingly defining actionable targets. Innate immune activation, endothelial injury, and ischemia–reperfusion injury, remain key barriers to durable xenograft function; nonetheless, tolerance‐focused approaches such as thymokidneys offer encouraging signals of donor‐specific hyporesponsiveness and immunosuppression minimization As new WHO guidance is developed, these advances collectively support a shift toward multi‐institutional trials, with clearer expectations for safety, reproducibility, and durability.
